# Assessment of bone turnover markers and DXA parameters to predict bone metastasis progression during zoledronate treatment: a single-center experience

**DOI:** 10.1007/s10238-023-01280-1

**Published:** 2024-01-19

**Authors:** Stella D’Oronzo, Mauro Cives, Eleonora Lauricella, Stefania Stucci, Antonella Centonza, Marica Gentile, Carmela Ostuni, Camillo Porta

**Affiliations:** 1 Interdisciplinary Department of Medicine, University of Bari Aldo Moro, Bari, Italy; 2 Division of Medical Oncology, A.O.U. Consorziale Policlinico Di Bari, Bari, Italy; 3 Unit of Oncology, Fondazione IRCCS “Casa Sollievo Della Sofferenza”, San Giovanni Rotondo, Italy; 4 Oncology Unit of National Institute of Gastroenterology - IRCCS “Saverio de Bellis”, Research Hospital Castellana Grotte, Bari, Italy

**Keywords:** Bone metastasis, Bone turnover markers, DXA, Zoledronate

## Abstract

Bone metastases (BM) are a serious cancer complication, potentially causing substantial morbidity. Among the clinical issues related to BM, there is the lack of specific tools for early diagnosis and prognosis. We explored whether combining bone turnover markers (BTM) with dual-energy X-ray absorptiometry (DXA) assessment could identify early BM progression and risk of skeletal-related events (SREs) during zoledronate treatment. Before the initiation of zoledronate (T0) and after six months of treatment (T1), serum levels of five BTM were measured, and patients (*N* = 47) underwent DXA evaluation. Standard radiological imaging was performed to assess bone tumor response to medical anti-cancer treatment. High tumor burden in bone correlated with higher serum CTX (*p* = 0.007) and NTX (*p* = 0.005) at baseline. Low concentrations of OPG at T0 predicted BM progression with a sensitivity and specificity of 63% and 77%, respectively, when a cutoff of 5.2 pmol/l was used; such a predictive meaning was stronger in patients with lytic BM (sensitivity: 88%, specificity: 80%; *p* = 0.0006). As for the risk of SREs, we observed an association between low baseline OC (*p* = 0.04) and OPG (*p* = 0.08) and the onset of any-time SREs, whereas an increase in OPG over time was associated with reduced risk of on-study events (*p* = 0.03). Moreover, a statistically significant correlation emerged between low baseline lumbar T-score and femur BMD and on-study SREs (*p* < 0.001 in both instances). These findings suggest that addition of DXA to BTM dosage could help stratifying the risk of SREs at the time of BM diagnosis but does not enhance our capability of detecting bone progression, during zoledronate treatment.

## Introduction

Bone metastases (BM) are a serious complication of several late-stage malignancies. In particular, breast and prostate cancers, which account for the majority of tumors in adult females and males, respectively, exhibit a marked tendency to colonize the skeleton, with up to 65–75% of patients experiencing bone disease when in stage IV [1]. In addition, a not negligible proportion of subjects suffering from thyroid, lung and renal cancer may develop BM during the course of their disease [2, 3].

Depending on the primary tumor and according to the radiographic features of the lesions, BM can be classified as either osteolytic or osteoblastic. When bone resorption prevails, as often observed in lung and renal cell carcinoma patients, focal bone destruction occurs, leading to the establishment of “lytic” lesions. On the other hand, BM characterized by enhanced osteoblastic activity, as in prostate cancer, appear osteosclerotic (also known as “osteoblastic”) [4]. However, even if one component seems to prevail over the other, bone resorption and osteogenesis are usually both accelerated within BM, and mixed lesions can be observed, especially in metastatic breast cancer patients [4].

Due to the considerable morbidity of skeletal metastases, their timely diagnosis and periodic monitoring are crucial. Current guidelines define plain X-ray, computerized tomography (CT) and radionuclide bone scan as the gold standard techniques for BM detection [5], whereas the interest toward whole-body magnetic resonance imaging (MRI) and positron emission tomography (PET) scan with different radiotracers is increasing [5–7]. However, rather than depicting the cancer cell foci, these techniques show the stromal reaction within the bone marrow, for which their sensitivity in detecting early-stage BM may be poor. In addition, skeletal lesions change during anti-cancer and anti-resorptive treatments, further complicating their monitoring over time [2].

In an attempt to overcome the limitations of current imaging techniques, which also include radiation exposure, economic burden for national health systems and/or financial toxicity for individual patients, few studies attempted to evaluate the role of dual-energy X-ray absorptiometry (DXA) in monitoring BM response to anti-cancer treatment, with promising results [8, 9].

Several efforts have also been made to evaluate the potential role of bone turnover markers (BTM), which are susceptible to non-invasive measurement in blood and urine [10–12], as a surrogate for radiological imaging [12–15], and/or as prognostic biomarkers in patients with metastatic bone disease [16–20]. However, conflicting results emerged from such studies, with high inter- and intra-individual variability representing a substantial limitation to their routine use [11].

In this single-center experience, we assessed whether the coupled use of DXA and BTM measurement could enable early identification of BM progression in a heterogeneous cohort of patients with skeletal metastases from solid malignancies receiving zoledronate during the anti-tumor treatment.

## Methods

### Patients

We enrolled patients with histologically confirmed solid malignancies and diagnosis of BM, attending the Medical Oncology Division of the University Hospital “Policlinico of Bari” (Bari, Italy). Patients were consecutively enrolled after signing written informed consent. Eligibility criteria included the presence of radiologically confirmed BM in patients aged ≥ 18 years, Eastern Cooperative Oncology Group-Performance Status (ECOG-PS) 0–2, adequate renal function (estimated glomerular filtration rate, eGFR ≥ 60 ml/min; serum creatinine ≤ 3 mg/dl) and a life expectancy ≥ 6 months. Neither prior anti-resorptive treatments nor previous hormone therapies were allowed. Further exclusion criteria included a diagnosis of active autoimmune disease that required steroids in the past two years, severe liver dysfunction, second malignancies as well as any contraindication for anti-cancer or bisphosphonate treatment. Clinical and pathological data from all patients were collected and recorded in an anonymized form. All enrolled patients received anti-cancer therapy, at the discretion of the treating physician, and 4 mg intravenous zoledronate every 4 weeks plus daily supplementation with calcium and vitamin D, according to current guidelines [5, 21].

### Radiological imaging

In agreement with current guidelines for BM diagnosis [5, 21], a full-body CT and a bone scan were performed before (T0) and 6 months after the initiation of zoledronate (T1); when deemed necessary by the clinician, a whole-body PET-CT scan with ^18^F-fluorodeoxyglucose (^18^F-FDG) or a spine MRI were also performed. Bone tumor response was assessed according to the MD Anderson (MDA) criteria [22, 23].

### BTM measurement

Blood samples were collected at T0 and T1 from all patients to measure serum levels of five BTM, namely bone alkaline phosphatase (BALP), C- and N-terminal telopeptides of type I collagen (CTX and NTX, respectively), osteocalcin (OC) and osteoprotegerin (OPG). All samples were collected in the early morning, after an overnight fast. BALP, CTX and OC levels were measured by using a chemiluminescence immunoassay, as described [15, 17, 24, 25], while NTX and OPG were dosed through an immunoenzymatic assay (ELISA) [25, 26]. Assessment of all BTM was performed in a CLIA-certified laboratory.

### DXA scan

In addition to standard radiological imaging, at both T0 and T1 all enrolled patients underwent densitometric evaluation by using a GE Lunar iDXA system (GE Healthcare, Chicago, Illinois, USA). The iDXA unit was checked daily using a calibration standard, to ensure that the instrument was operating within the manufacturer’s specifications. A trained operator made all scans following the manufacturer’s manual and data were analyzed by using the software ncore (version 13.60). Bone mineral density (BMD: g/cm^2^) and T-score were assessed at both lumbar spine (L1-L4) and femur neck, as described [27, 28]. In addition, for each patient, a target metastatic bone lesion was arbitrarily selected for densitometric evaluation, after manual determination of the region of interest (ROI) [29, 30].

### Statistical analyses

Descriptive statistics were used for patient demographics. The association between dichotomous variables was evaluated by Fisher’s test or ANOVA, as appropriate. The Wilcoxon-matched pairs signed rank test was used to evaluate over time changes of continuous variables. Receiver operating characteristics (ROC) curve analysis was used to set the optimal cutoff point for possible predictors of bone disease progression. DeLong’s test was used to compare the area under the ROC curves (AUC). Overall survival (OS) was measured from date of initial diagnosis until death from any cause or last known follow-up. Time-to-event functions were estimated by the Kaplan–Meier method and compared using the log-rank test. Exact 95% confidence intervals (CI) were calculated for each proportion of interest. All tests were two-sided, and statistical significance was declared at a P value of 0.05 or less. Statistical analysis was conducted using MedCalc statistical software 12.7 (MedCalc Software bvba, Ostend, Belgium).

## Results

### Demographics and tumor characteristics

Demographic variables and clinical-pathological characteristics of the 47 patients included in the study are summarized in Table 1. The majority of the patients harbored malignancies of the breast (*n* = 15), lung (*n* = 8) and prostate (*n* = 7), and the median age at cancer diagnosis was 63 years (range 32–89 years). All female patients were post-menopausal at the time of enrollment. In 53% of patients, > 3 BM were revealed by imaging, while the remaining 47% presented with ≤ 3 skeletal lesions. The radiological pattern of BM was shown to be osteolytic, osteoblastic or mixed in 38.3%, 36.2% and 25.5% of patients, respectively, and the lesions were primarily located in the axial skeleton (92% of cases). Skeletal-related events (SREs) occurred in 42% of patients during the course of the disease (any-time SREs), whereas on-study SREs (namely the SREs occurring in the T0-T1 interval) were reported in 8 out of 47 (17%) enrolled subjects. Most of the patients (57%) had not received a prior systemic anti-cancer treatment at study enrollment. The proportion of arbitrarily chosen lytic or sclerotic DXA target lesions was similar (49% vs 51%), and most of them were located in the axial skeleton (77%).

**Table 1:** Patient demographics, primary tumor and skeletal metastasis characteristics

Characteristics	No. of patients	%
	(*n* = 47)	
Age at diagnosis (years)		
Median	63	
Range	32–89	
Gender		
Male	23	48.9
Female	24	51.1
BMI		
Median	25.7	
Range	17.2–37.9	
Primary tumor site		
Breast cancer	15	31.9
Lung cancer	8	17
Prostate cancer	7	14.9
Kidney cancer	5	10.6
Others	12	25.5
Gastrointestinal tract cancers	5	
Head & neck tumors	4	
Melanoma	2	
Bladder cancer	1	
Number of skeletal metastases		
≤ 3	22	46.8
> 3	25	53.2
Type of skeletal metastases		
Lytic	18	38.3
Sclerotic	17	36.2
Mixed	12	25.5
Localization of skeletal metastases		
Axial	17	36.2
Appendicular	4	8.5
Both	26	55.3
SREs during the course of the disease		
No	27	57.4
Yes	20	42.6
Pathological fracture	4	8.5
Need for RT	12	25.5
Need for orthopedic surgery	4	8.5
First on-study SREs		
No	39	83
Yes	8	17
Pathological fracture	2	4.2
Need for RT	5	10.6
Need for orthopedic surgery	1	2.1
Previous systemic anti-cancer treatment		
No	27	57.4
Yes	20	42.6
Platinum-based regimen	4	8.5
Taxane-based regimen	4	8.5
Others	5	10.6
Unknown	2	4.2
Localization of DXA target lesion		
Thoracic vertebra	13	27.6
Lumbar vertebra	15	31.9
Pelvic bone	8	17
Femur	8	17
Others	3	6.4
Type of DXA target lesion		
Lytic	23	49
Sclerotic	24	51

*BMI* body mass index, *DXA* dual-energy X-ray absorptiometry, *RT* radiotherapy, *SREs* skeletal-related events

### Association between baseline BTMs, DXA parameters and BM characteristics

**Fig. 1:**
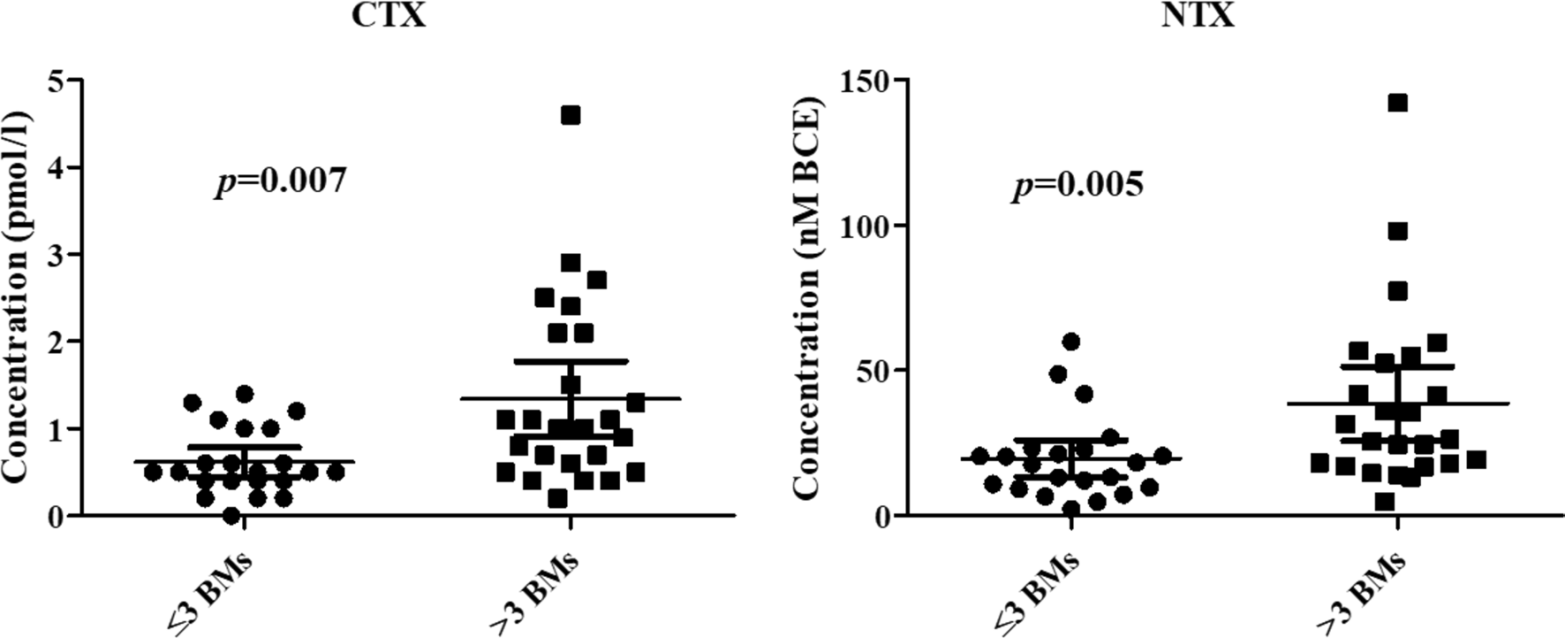
Serum concentrations of CTX and NTX at baseline varied according to the number of BM. Baseline serum concentrations of both CTX and NTX were significantly higher in patients with more extensive skeletal colonization

We explored the association between baseline levels of BTMs and the number (≤ 3 vs > 3), type (lytic vs sclerotic vs mixed) and site (axial vs appendicular vs both) of skeletal metastases. As shown in Fig. 1, patients with more than 3 skeletal lesions had significantly higher concentrations of CTX (*p* = 0.007) and NTX (*p* = 0.005) as compared with subjects harboring ≤ 3 skeletal lesions. No correlation was observed between baseline concentrations of BTMs and the remaining explored features. We next evaluated the association between key DXA parameters including lumbar, femur and target lesion BMD or T-score, and BM characteristics. As expected, the BMD of osteolytic target lesions was significantly lower than that of osteoblastic metastases (*p* = 0.01). All other DXA parameters were not significantly associated with the remaining clinical-radiological features.

### Over time change of BTMs and DXA parameters

**Fig. 2:**
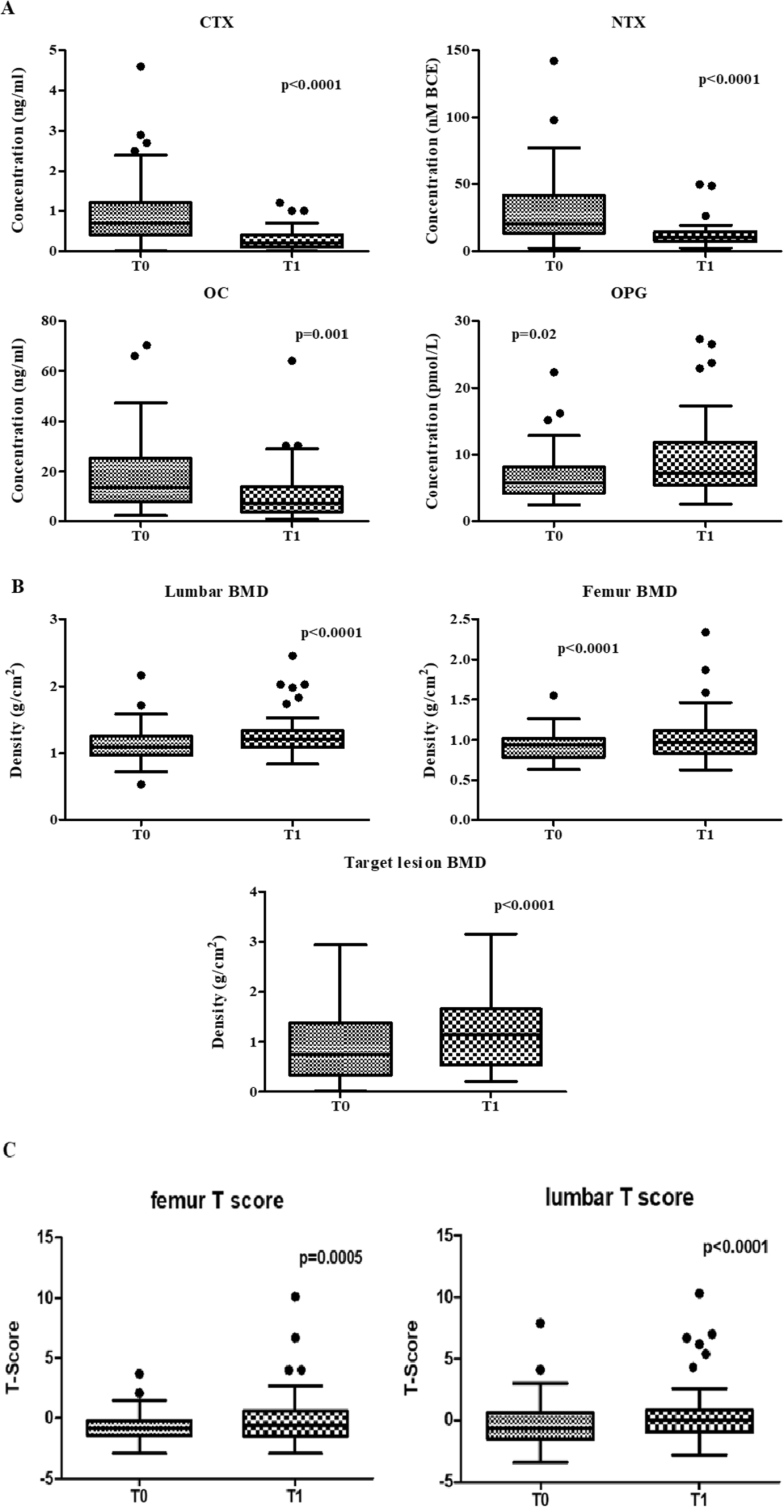
BTMs and DXA parameters changed over time during anti-cancer and bisphosphonate treatment. **A** During the first 6 months of treatment with zoledronate (in addition to anti-cancer treatment), we observed a significant decrease in both CTX and NTX, as markers of bone resorption (*p* < 0.0001 in both instances). Among markers of bone formation, OC was also found significantly reduced (*p* = 0.001) whereas OPG turned out increased (*p* = 0.02). **B** Significant improvements of both lumbar and femur BMD were registered from T0 to T1, associated with increased BMD in the target bone lesion (*p* < 0.0001 in all instances). **C** T-scores calculated at both skeletal sites (femur and lumbar spine) significantly increased after bisphosphonate treatment

We then measured the change induced on BTM and DXA parameters by the administration of zoledronate during anti-cancer treatment, over a 6-month time frame. As depicted in Fig. 2A, we found a significant decrease in CTX (*p* < 0.0001), NTX (*p* < 0.0001) and OC levels (*p* = 0.01) over time, in the presence of a significant increase in OPG serum concentration (*p* = 0.02). Moreover, lumbar (*p* < 0.0001), femur (*p* < 0.0001) and target lesion BMD (*p* < 0.0001) turned out all significantly increased after 6 months of zoledronate treatment as compared with baseline (Fig. 2B). A similar change was also noted in both lumbar (*p* < 0.0001) and femur T-score (*p* = 0.0005) (Fig. 2C).

### Prediction of skeletal progression by BTM and DXA parameters

We next investigated the ability of either baseline BTM/DXA features or their over time changes in predicting skeletal progression during the first 6 months of zoledronate treatment. At T1, 19 patients experienced disease progression in bone, whereas the remaining exhibited stable disease (*N* = 22) or response to treatment (*N* = 6).

**Fig. 3:**
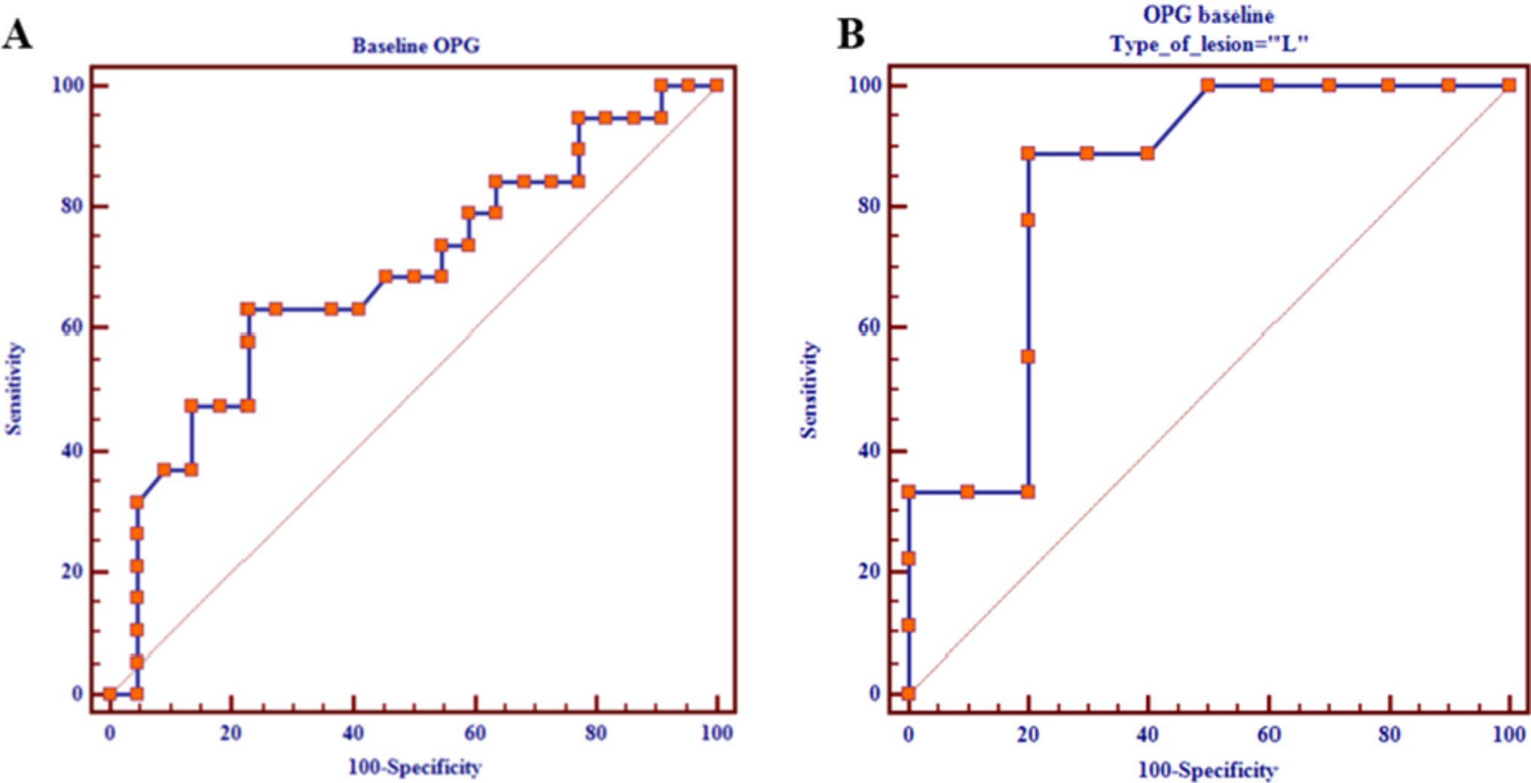
Baseline OPG predicts early disease progression in bone. **A** Low serum concentration of OPG at baseline predicts bone disease progression (*p* = 0.03) with a sensitivity of 63% and specificity of 77% when a cutoff of 5.2 pmol/l is used. **B** The predictive effect of OPG is particularly marked in patients with lytic BM (*n* = 23), showing a sensitivity of 88% and a specificity of 80% (*p* = 0.0006)

Low baseline concentrations of OPG turned out able to predict bone disease progression (*p* = 0.03), showing a sensitivity and specificity of 63% and 77%, respectively, when a cutoff of 5.2 pmol/l was used (Fig. 3A). The predictive effect of OPG (same cutoff value) appeared particularly marked in patients harboring only lytic lesions (*n* = 23), in the presence of a sensitivity of 88% and a specificity of 80% (*p* = 0.0006) (Fig. 3B). No significant association was found between the remaining baseline BTM/DXA features and the skeletal disease progression. Similarly, no correlation was observed between over time changes of BTM concentrations and DXA parameters and bone tumor progression.

### Association between BTMs, DXA parameters and SREs

**Fig. 4:**
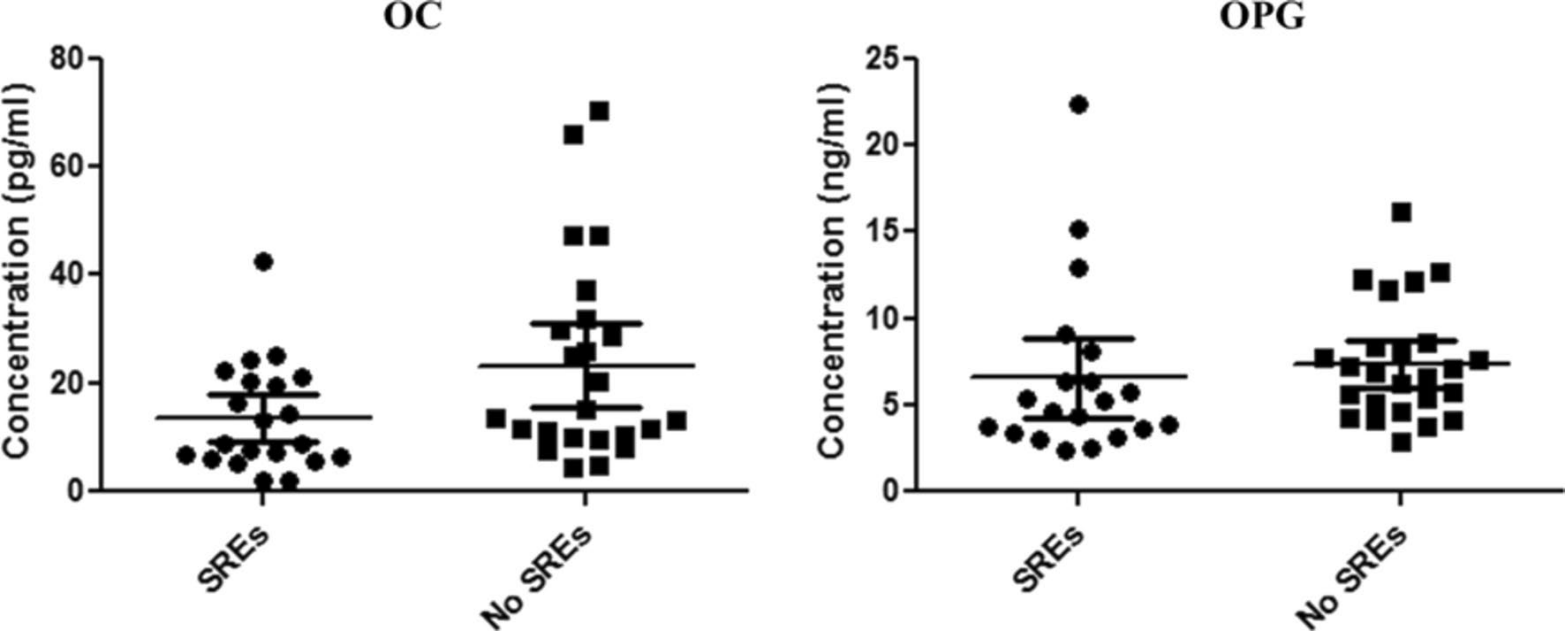
Association between BTM levels and onset of SREs. Low baseline concentrations of OC (*p* = 0.04) (on the left) and OPG (*p* = 0.08) (on the right) correlated with the onset of any-time SREs

We then evaluated the association between baseline BTM/DXA features, as well as BTM/DXA longitudinal changes, and SRE occurrence. We observed an association between low baseline concentrations of OC (*p* = 0.04) and OPG (*p* = 0.08) and the onset of any-time SREs (Fig. 4). Over time increase in OPG levels was associated with a reduced risk of on-study SREs (*p* = 0.03). With respect to DXA parameters, we observed a statistically significant correlation between low baseline lumbar T-score and femur BMD and on-study SREs (*p* < 0.001 in both instances), whereas such a correlation was not observed with femur T-score and lumbar BMD.

### Prognostic impact of BTMs and DXA parameters

In our cohort, the median overall survival (OS) was 40.3 months (95% CI 33.1–71.6 months; data not shown). When OS was evaluated from BM detection instead of cancer diagnosis, the median OS was 34.8 months (95% CI 25.1–47.2 months, data not shown). In patients experiencing SREs, median survival from first SRE was 33 months (95% CI 10–46.6 months). No significant association was noted between baseline BTM/DXA features or BTM/DXA longitudinal changes and OS.

## Discussion

BM are a common complication of solid tumors, accounting for substantial morbidity and mortality, as well as socioeconomic costs. One of the major clinical issues related to BM is the lack of specific biomarkers for early diagnosis and treatment monitoring [2, 31]. To our knowledge, this is the first study to assess the role of integrated BTMs and DXA scan in predicting the efficacy of zoledronate in terms of bone disease control and prevention of SREs in a heterogeneous and “real-world” cohort of cancer patients.

BTMs have been already investigated as potential biomarkers of BM, and a correlation between BTMs and the extent of metastatic bone disease as well as the risk of SREs has been described [32]. Consistent with prior observations [33–35], we found that baseline levels of the bone resorption markers CTX and NTX significantly correlated with the extent of skeletal disease in our cohort. While intra- and inter-individual variability negatively affect the reliability of urine BTMs, thus limiting their routine clinical use, we note that only serum measurements were used in our study in order to overcome the need of creatinine correction [12] and minimize the assay variability [26]. In agreement with previous reports [13, 25], a significant reduction of CTX and NTX was observed during the first 6 months of zoledronate treatment. However, no association was found between longitudinal changes of these markers (reduction or increase) and skeletal progression by MDA criteria or SRE occurrence.

Among osteoblast-derived proteins, OC and OPG showed an opposite direction in terms of over time change during bisphosphonate treatment in our study. Nevertheless, both the decrease in OC and the increase in OPG are not surprising findings. OC is the most abundant non-collagenous protein in bone and is released into systemic circulation during bone resorption [36], a process known to be negatively modulated by zoledronate. On the other hand, OPG is a marker of osteoblast differentiation [37], which is fostered by bisphosphonates [38, 39]. In our cohort, low levels of both OC and OPG predicted early skeletal progression, especially in patients with lytic BM. On the other hand, a progressive increase in OPG concentration during zoledronate treatment predicted a reduced probability of SREs. To the best of our knowledge, this is the first evidence of such a clinical value of OPG and deserves further investigation in a wider patient cohort.

The role of DXA in predicting BM response to zoledronate has been poorly investigated so far. Sporadic reports [40–42] have described the ability of DXA scan (performed to rule out osteoporosis in patients without an established diagnosis of cancer or in the setting of cancer treatment-induced bone loss, CTIBL) to reveal the presence of skeletal lesions, subsequently diagnosed as BM. In a previous report, Shapiro et al. [8] prospectively evaluated the role of DXA in monitoring BM response to anti-cancer treatment in 9 patients with breast cancer. The over time change of skeletal metastasis BMD was shown to correlate with the findings from standard imaging modalities, such as X-rays and CT scan, and a significant association was observed between BMD increase and response to treatment.

In line with prior evidence [9, 43–45], the BMD of osteolytic lesions was significantly lower than that of osteosclerotic metastases in our study. Moreover, as expected, lumbar, femur and target lesion BMD as well as lumbar and femur T-score significantly increased during zoledronate treatment. While longitudinal changes of either BMD or T-score failed to predict BM progression or SRE occurrence, the presence of two abnormal DXA parameters at baseline (low lumbar T-score and low femur BMD) was associated with higher risk of on-study SREs. Such a correlation suggests that a routine densitometric assessment of bone metastatic patients at baseline could be useful for stratifying their risk of skeletal complications and plan appropriate therapeutic strategies. Indeed, pre-existing osteopenic/osteoporotic conditions might further increase bone fragility in patients with BM.

It has to be noted that a variable (between 3 and 40%) degree of discordance between lumbar spine and femur DXA parameters has been described in the literature [46–48], due to physiological and anatomical differences, as well as potential artifactual and technical issues; this might explain why only two DXA parameters correlated, in our series, with the risk of SREs, while the remaining did not.

Although intriguing, these findings might have been biased by the heterogeneity of our “real-world” patient cohort and need further confirmation in wider, more homogeneous series.

Our analysis did not show any correlation between BTMs or densitometric parameters and OS, but the heterogeneity of the cohort, coupled with that of administered anti-tumor treatments, might have confounded our results.

Our work has other limitations. First, the small sample size limits the power of our analyses, hindering definitive conclusions on the role of BTMs and densitometric parameters in monitoring the activity of zoledronate. Second, the heterogeneity of the cohort (in terms of cancer diagnosis, tumor molecular features, age of the patients, smoking habits, administered anti-tumor treatments, etc.) may bias our results, introducing uncontrolled confounding factors.

Nevertheless, our cohort mirrors a “real-world” scenario, providing information that are directly applicable to the routine clinical practice. In addition, patients with a personal history of hormone treatment (which is a well-established modifier of bone turnover) [21] were excluded from our study to reduce the risk of pre-existing iatrogenic bone health alterations (i.e. CTIBL). As for the hormone anti-cancer therapies administered between T0 and T1, instead, we did not expect them to have relevant impact on bone turnover during zoledronate treatment, since several clinical trials have described that up-front concomitant administration of the bisphosphonate reduces the risk of bone loss, even when the 6-monthly schedule of the drug is applied, as in the setting of CTIBL prevention [49–53]. Moreover, over time comparisons within the same patient should be less dependent on the aforementioned confounders, having an internal baseline control.

In conclusion, improvement of densitometric parameters or BTMs during the first 6 months of zoledronate treatment is not necessarily associated with a reduced risk of bone disease progression. Low baseline concentrations of OC and OPG as well as low baseline T-score and BMD might predict the occurrence of SREs. Therefore, integration of BTM dosage and DXA evaluation at diagnosis of BM may be helpful in better stratifying the risk of SREs. Future studies in larger, more homogeneous cohorts of patients should evaluate whether the increase in OPG levels during zoledronate therapy may truly predict a reduced risk of SREs.

## References

[1] Hofbauer LC, Bozec A, Rauner M, Jakob F, Perner S, Pantel K. Novel approaches to target the microenvironment of bone metastasis. Nat Rev Clin Oncol. 2021;18(8):488–505. 33875860 10.1038/s41571-021-00499-9

[2] D’ Oronzo S, Coleman R, Brown J, Silvestris F. Metastatic bone disease: pathogenesis and therapeutic options: up-date on bone metastasis management. J Bone Oncol. 2018. 10.1016/j.jbo.2018.10.004. 30937279 10.1016/j.jbo.2018.10.004 PMC6429006

[3] Macedo F, Ladeira K, Pinho F, et al. Bone metastases: an overview. Oncol Rev. 2017;11(1):321. 28584570 10.4081/oncol.2017.321 PMC5444408

[4] Coleman RE. Clinical features of metastatic bone disease and risk of skeletal morbidity. Clin Cancer Res. 2006;12(20 Pt 2):6243s–9s. 17062708 10.1158/1078-0432.CCR-06-0931

[5] Coleman R, Hadji P, Body JJ, et al. Bone health in cancer: ESMO clinical practice guidelines. Ann Oncol. 2020;31(12):1650–63. 32801018 10.1016/j.annonc.2020.07.019

[6] Broski SM, Young JR, Kendi AT, Subramaniam RM. Skeletal metastasis evaluation: value and impact of PET/computed tomography on diagnosis, management and prognosis. PET Clin. 2019;14(1):103–20. 30420213 10.1016/j.cpet.2018.08.006

[7] Oprea-Lager DE, Cysouw MCF, Boellaard R, et al. Bone metastases are measurable: the role of whole-body MRI and positron emission tomography. Front Oncol. 2021;11: 772530. 34869009 10.3389/fonc.2021.772530 PMC8640187

[8] Shapiro CL, Keating J, Angell JE, et al. Monitoring therapeutic response in skeletal metastases using dual-energy x-ray absorptiometry: a prospective feasibility study in breast cancer patients. Cancer Invest. 1999;17(8):566–74. 10592763 10.3109/07357909909032841

[9] Perk H, Yildiz M, Kosar A, Cerci S, Soyupek F, Ozorak A, Dilmen C. Correlation between BMD and bone scintigraphy in patients with prostate cancer. Urol Oncol. 2008;26(3):250–3. 18452814 10.1016/j.urolonc.2007.05.027

[10] Gentile M, Centonza A, Lovero D, et al. Application of “omics” sciences to the prediction of bone metastases from breast cancer: state of the art. J Bone Oncol. 2020;26: 100337. 33240786 10.1016/j.jbo.2020.100337 PMC7672315

[11] D’Oronzo S, Brown J, Coleman R. The role of biomarkers in the management of bone-homing malignancies. J Bone Oncol. 2017;9:1–9. 28948139 10.1016/j.jbo.2017.09.001 PMC5602513

[12] Coleman R, Costa L, Saad F, et al. Consensus on the utility of bone markers in the malignant bone disease setting. Crit Rev Oncol Hematol. 2011;80(3):411–32. 21411334 10.1016/j.critrevonc.2011.02.005

[13] Costa L, Demers LM, Gouveia-Oliveira A, et al. Prospective evaluation of the peptide-bound collagen type I cross-links N-telopeptide and C-telopeptide in predicting bone metastases status. J Clin Oncol. 2002;20(3):850–6. 11821470 10.1200/JCO.2002.20.3.850

[14] Santini D, Cinieri S, Gasparro D, et al. Effects of abiraterone acetate plus prednisone on bone turnover markers in chemotherapy-naïve mCRPC patients after ADT failure: a prospective analysis of the italian real-world study ABITUDE. J Bone Oncol. 2020;26: 100341. 33425672 10.1016/j.jbo.2020.100341 PMC7779770

[15] Windrichova J, Kucera R, Fuchsova R, et al. An assessment of novel biomarkers in bone metastatic disease using multiplex measurement and multivariate analysis. Technol Cancer Res Treat. 2018;17:1533033818807466. 30343636 10.1177/1533033818807466 PMC6198393

[16] Lara PN Jr, Plets M, Tangen C, et al. Bone turnover biomarkers identify unique prognostic risk groups in men with castration resistant prostate cancer and skeletal metastases: Results from SWOG S0421. Cancer Treat Res Commun. 2018;16:18–23. 31298998 10.1016/j.ctarc.2018.04.005 PMC6628720

[17] Urakawa H, Ando Y, Hase T, et al. Clinical value of serum bone resorption markers for predicting clinical outcomes after use of bone modifying agents in metastatic bone tumors: a prospective cohort study. Int J Cancer. 2020;146(12):3504–15. 31846063 10.1002/ijc.32836

[18] Jiang Z, Tang ET, Li C, et al. What is the relationship between bone turnover markers and skeletal-related events in patients with bone metastases from solid tumors and in patients with multiple myeloma? A systematic review and meta-regression analysis. Bone Rep. 2020;12: 100272. 32420416 10.1016/j.bonr.2020.100272 PMC7215099

[19] Lipton A, Cook R, Brown J, Body JJ, Smith M, Coleman R. Skeletal-related events and clinical outcomes in patients with bone metastases and normal levels of osteolysis: exploratory analyses. Clin Oncol (R Coll Radiol). 2013;25(4):217–26. 23219232 10.1016/j.clon.2012.11.004

[20] Shizuku M, Shibata M, Okumura M, Takeuchi D, Kikumori T, Mizuno Y. Utility of urinary type I collagen cross-linked N-telopeptide as a prognostic indicator in breast cancer patients with bone metastases. Breast Cancer. 2020;27(6):1065–71. 32415556 10.1007/s12282-020-01109-9

[21] Coleman R, Body JJ, Aapro M, Hadji P, Herrstedt J. Bone health in cancer patients: ESMO clinical practice guidelines. Ann Oncol. 2014;25(Supplement 3):iii124–37. 24782453 10.1093/annonc/mdu103

[22] Hayashi N, Costelloe CM, Hamaoka T, et al. A prospective study of bone tumor response assessment in metastatic breast cancer. Clin Breast Cancer. 2013;13(1):24–30. 23098575 10.1016/j.clbc.2012.09.004 PMC3863546

[23] Costelloe CM, Chuang HH, Madewell JE, Ueno NT. Cancer response criteria and bone metastases: RECIST 1.1, MDA and PERCIST. J Cancer. 2010;1:80–92. 20842228 10.7150/jca.1.80 PMC2938069

[24] Han S, Xue Y, Zhang J, et al. A chemiluminescent immunoassay for osteocalcin in human serum and a solution to the “hook effect.” J Anal Methods Chem. 2020;2020:8891437. 33376621 10.1155/2020/8891437 PMC7744234

[25] Nishimukai A, Higuchi T, Ozawa H, et al. Different patterns of change in bone turnover markers during treatment with bone-modifying agents for breast cancer patients with bone metastases. Breast Cancer. 2017;24(2):245–53. 27040403 10.1007/s12282-016-0695-2

[26] Jung K, Lein M. Bone turnover markers in serum and urine as diagnostic, prognostic and monitoring biomarkers of bone metastasis. Biochem Biophys Acta. 2014;1846:425–38. 25220832 10.1016/j.bbcan.2014.09.001

[27] Watts NB. Fundamentals and pitfalls of bone densitometry using dual-energy X-ray absorptiometry (DXA). Osteoporos Int. 2004;15:847–54. 15322740 10.1007/s00198-004-1681-7

[28] Ward RJ, Roberts CC, Bencardino JT, et al. ACR appropriateness criteria^®^ osteoporosis and bone mineral density. J Am Coll Radiol. 2017;14(5S):S189–202. 28473075 10.1016/j.jacr.2017.02.018

[29] Lohman M, Tallroth K, Kettunen JA, Marttinen MT. Reproducibility of dual-energy x-ray absorptiometry total and regional body composition measurements using different scanning positions and definitions of regions. Metabolism. 2009;58(11):1663–8. 19632696 10.1016/j.metabol.2009.05.023

[30] Burkhart TA, Arthurs KL, Andrews DM. Manual segmentation of DXA scan images results in reliable upper and lower extremity soft and rigid tissue mass estimates. J Biomech. 2009;42(8):1138–42. 19356763 10.1016/j.jbiomech.2009.02.017

[31] Song MK, Park SI, Cho SW. Circulating biomarkers for diagnosis and therapeutic monitoring in bone metastasis. J Bone Miner Metab. 2023;41(3):337–44. 36729305 10.1007/s00774-022-01396-6

[32] Iuliani M, Simonetti S, Ribelli G, et al. Current and emerging biomarkers predicting bone metastasis development. Front Oncol. 2020;10:789. 32582538 10.3389/fonc.2020.00789 PMC7283490

[33] Koizumi M, Takahashi S, Ogata E. Comparison of serum bone resorption markers in the diagnosis of skeletal metastasis. Anticancer Res. 2003;23(5b):4095–9. 14666607

[34] Zhao H, Han KL, Wang ZY, et al. Value of C-telopeptide-cross-linked Type I collagen, osteocalcin, bone-specific alkaline phosphatase and procollagen Type I N-terminal propeptide in the diagnosis and prognosis of bone metastasis in patients with malignant tumors. Med Sci Monit. 2011;17(11):CR626–33. 22037741 10.12659/MSM.882047 PMC3539492

[35] Li L, Shen X, Liang Y, Li B, Si Y, Ma R. N-telopeptide as a potential diagnostic and prognostic marker for bone metastasis in human cancers: a meta-analysis. Heliyon. 2023;9(5): e15980. 37215848 10.1016/j.heliyon.2023.e15980 PMC10199183

[36] Martiniakova M, Mondockova V, Biro R, et al. The link between bone-derived factors osteocalcin, fibroblast growth factor 23, sclerostin, lipocalin 2 and tumor bone metastasis. Front Endocrinol (Lausanne). 2023;14:1113547. 36926025 10.3389/fendo.2023.1113547 PMC10012867

[37] Gori F, Hofbauer LC, Dunstan CR, Spelsberg TC, Khosla S, Riggs BL. The expression of osteoprotegerin and RANK ligand and the support of osteoclast formation by stromal-osteoblast lineage cells is developmentally regulated. Endocrinology. 2000;141(12):4768–76. 11108292 10.1210/endo.141.12.7840

[38] Viereck V, Emons G, Lauck V, et al. Bisphosphonates pamidronate and zoledronic acid stimulate osteoprotegerin production by primary human osteoblasts. Biochem Biophys Res Commun. 2002;291(3):680–6. 11855844 10.1006/bbrc.2002.6510

[39] Mercatali L, Ricci M, Scarpi E, et al. RANK/RANK-L/OPG in patients with bone metastases treated with anticancer agents and zoledronic acid: a prospective study. Int J Mol Sci. 2013;14(6):10683–93. 23702841 10.3390/ijms140610683 PMC3709696

[40] Fan P, Wang Q, Lu C, Chen D. Generalized high bone mineral density on bone density scanning: a case of gastric carcinoma with bone metastasis. Postgrad Med. 2017;129(2):299–303. 27849427 10.1080/00325481.2017.1261607

[41] Ustün N, Ustün I, Ozgür T, et al. Diffuse osteosclerosis in a patient with prostate cancer. Osteoporos Int. 2014;25(3):1181–5. 24136106 10.1007/s00198-013-2545-9

[42] Forbes V, Taxel P. Onset of asymptomatic skeletal metastatic disease seen on DXA. AACE Clin Case Rep. 2018;4(6):e472–5.

[43] Chang CH, Tsai CS, Jim YF, Wu HC, Lin CC, Kao A. Lumbar bone mineral density in prostate cancer patients with bone metastases. Endocr Res. 2003;29(2):177–82. 12856804 10.1081/erc-120022298

[44] Smith GL, Doherty AP, Banks LM, et al. Dual X-ray absorptiometry detects disease- and treatment-related alterations of bone density in prostate cancer patients. Clin Exp Metastasis. 2000;18(5):385–90. 11467770 10.1023/a:1010991213842

[45] Tanaka H, Furukawa Y, Fukunaga K, Fukunaga M. Bone mineral density for patients with bone metastasis of prostate cancer: a preliminary report. Adv Exp Med Biol. 1992;324:217–31. 1492619 10.1007/978-1-4615-3398-6_25

[46] Alarkawi D, Bliuc D, Nguyen TV, Eisman JA, Center JR. Contribution of lumbar spine BMD to fracture risk in individuals with T-score discordance. J Bone Miner Res. 2016;31(2):274–80. 26241926 10.1002/jbmr.2611

[47] Lee KH, Park JW, Kim S, Lee GY, Park SB, Yang DB, Ha YC. Prevalence, clinical implication, and cause of spine hip discordance in elderly patients with fragility hip fracture. J Bone Metab. 2022;29(1):51–7. 35325983 10.11005/jbm.2022.29.1.51 PMC8948489

[48] Chiang MH, Jang YC, Chen YP, Chan WP, Lin YC, Huang SW, Kuo YJ. T-score discordance between hip and lumbar spine: risk factors and clinical implications. Ther Adv Musculoskelet Dis. 2023;15:1759720X231177147. 37359176 10.1177/1759720X231177147 PMC10286209

[49] Brufsky AM, Harker WG, Beck JT, et al. Final 5-year results of Z-FAST trial: adjuvant zoledronic acid maintains bone mass in postmenopausal breast cancer patients receiving letrozole. Cancer. 2012;118(5):1192–201. 21987386 10.1002/cncr.26313

[50] Llombart A, Frassoldati A, Paija O, et al. Immediate administration of zoledronic acid reduces aromatase inhibitor-associated bone loss in postmenopausal women with early breast cancer: 12-month analysis of the E-ZO-FAST trial. Clin Breast Cancer. 2012;12(1):40–8. 22014381 10.1016/j.clbc.2011.08.002

[51] Coleman R, de Boer R, Eidtmann H, et al. Zoledronic acid (zoledronate) for postmenopausal women with early breast cancer receiving adjuvant letrozole (ZO-FAST study): final 60-month results. Ann Oncol. 2013;24(2):398–405. 23047045 10.1093/annonc/mds277

[52] Michaelson MD, Kaufman DS, Lee H, et al. Randomized controlled trial of annual zoledronic acid to prevent gonadotropin-releasing hormone agonist-induced bone loss in men with prostate cancer. J Clin Oncol. 2007;25(9):1038–42. 17369566 10.1200/JCO.2006.07.3361 PMC3047397

[53] Smith MR, Eastham J, Gleason DM, Shasha D, Tchekmedyian S, Zinner N. Randomized controlled trial of zoledronic acid to prevent bone loss in men receiving androgen deprivation therapy for non metastatic prostate cancer. J Urol. 2003;169(6):2008–12. 12771706 10.1097/01.ju.0000063820.94994.95

